# Supplementary material for: Modeling of mitochondrial genetic polymorphisms reveals induction of heteroplasmy by pleiotropic disease locus 10398A>G
Source: Sci Rep. 2023 Jun 27;13:10405. doi: 10.1038/s41598-023-37541-y (PMC10300032; doi:10.1038/s41598-023-37541-y)
Supplement: Supplementary file 1 — Supplementary Figures. [file 41598_2023_37541_MOESM1_ESM.docx]

# Figure S1


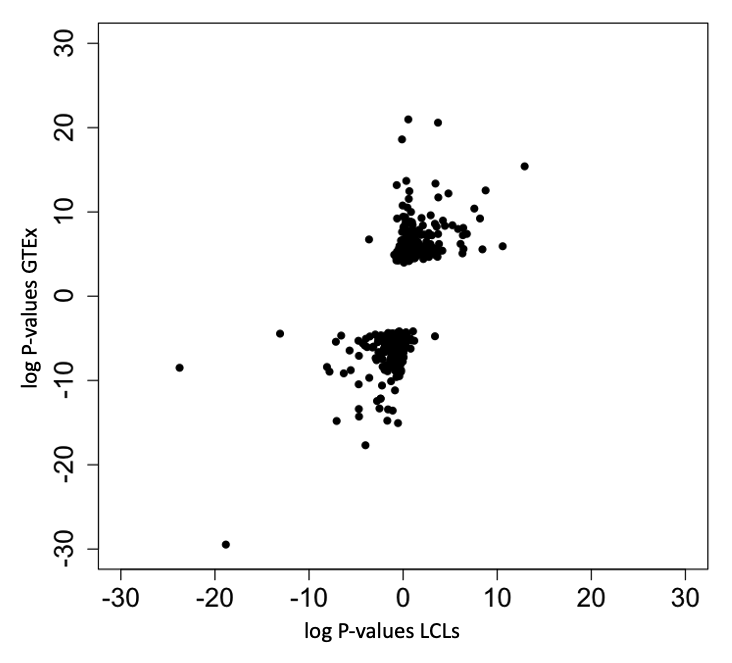


Comparing the log P-values of gene expression QTLs between the data obtained from our LCL samples versus LCL data obtained from GTEx. Negative log P-values indicate genes annotated as having lower gene expression with the effect allele whereas positive log P-values indicate genes annotated as having higher gene expression with the effect allele.

# Figure S2

Sanger sequencing results for all 60 samples to verify the mitochondria allele status for each sample. The fragment sequence depicted for each sample is ACTGA**[A/G]**CCGAATT.


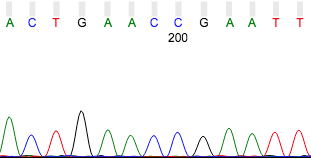


M1-MT1_FOR3 (Allele detected: A)


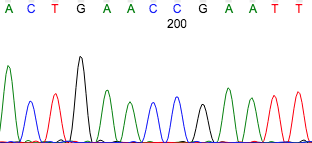


M2-MT1_FOR3 (Allele detected: A)


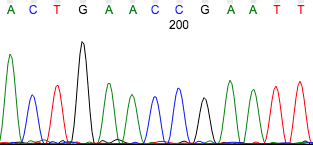


M3-MT1_FOR3 (Allele detected: A)


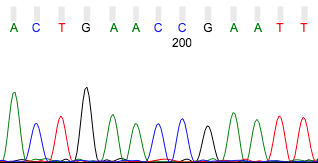


M4-MT1_FOR3 (Allele detected: A)


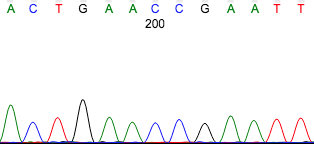


M5-MT1_FOR3 (Allele detected: A)


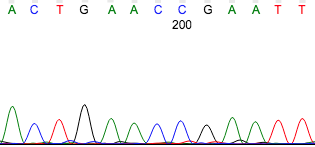


M6-MT1_FOR3 (Allele detected: A)


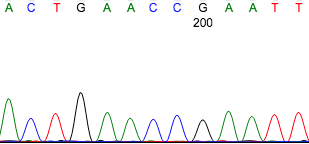


M7-MT1_FOR3 (Allele detected: A)


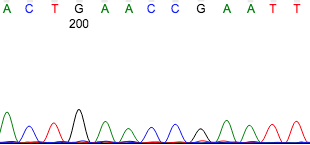


M8-MT1_FOR3 (Allele detected: A)


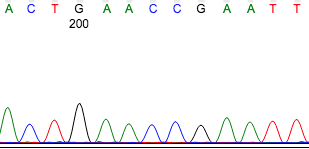


M9-MT1_FOR3 (Allele detected: A)


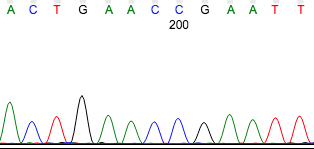


M10-MT1_FOR3 (Allele detected: A)


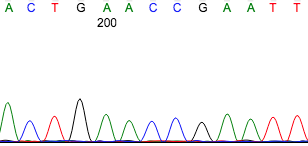


M11-MT1_FOR3 (Allele detected: A)


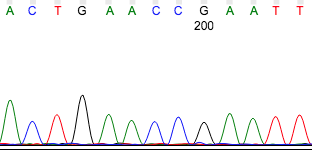


M12-MT1_FOR3 (Allele detected: A)


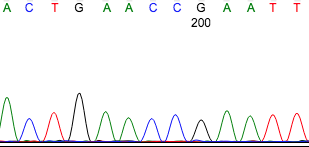


M13-MT1_FOR3 (Allele detected: A)


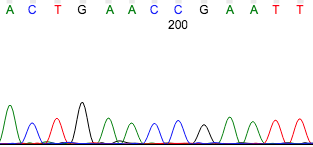


M14-MT1_FOR3 (Allele detected: A)


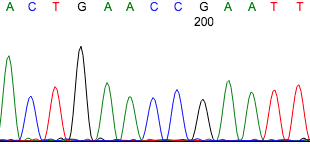


M15-MT1_FOR3 (Allele detected: A)


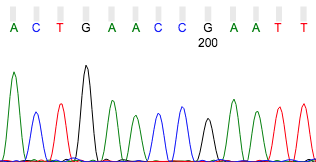


M16-MT1_FOR3 (Allele detected: A)


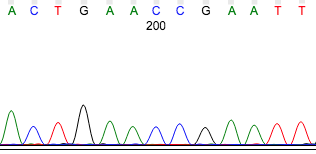


M17-MT1_FOR3 (Allele detected: A)


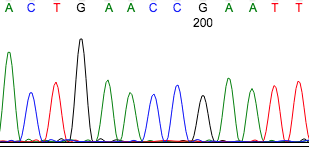


M18-MT1_FOR3 (Allele detected: A)


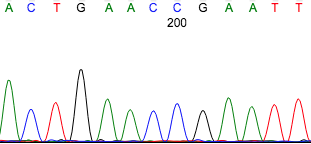


M19-MT1_FOR3 (Allele detected: A)


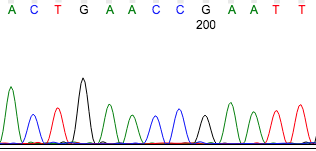


M20-MT1_FOR3 (Allele detected: A)


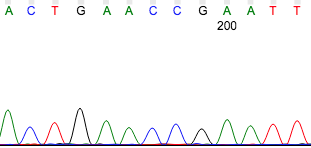


M21-MT1_FOR3 (Allele detected: A)


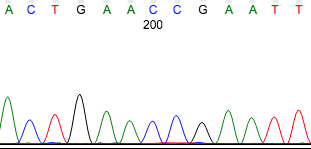


M22-MT1_FOR3_R (Allele detected: A)


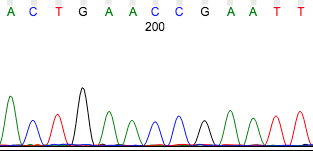


M23-MT1_FOR3 (Allele detected: A)


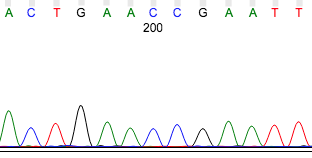


M24-MT1_FOR3 (Allele detected: A)


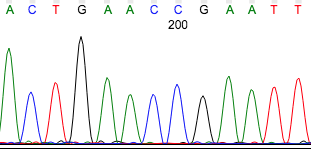


M25-MT1_FOR3 (Allele detected: A)


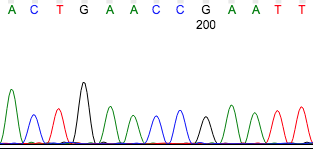


M26-MT1_FOR3 (Allele detected: A)


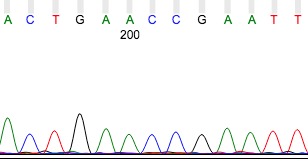


M27-MT1_FOR3 (Allele detected: A)


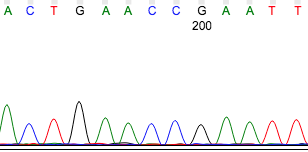


M28-MT1_FOR3 (Allele detected: A)


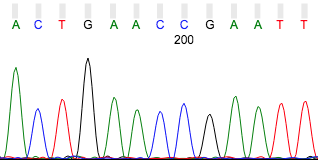


M29-MT1_FOR3 (Allele detected: A)


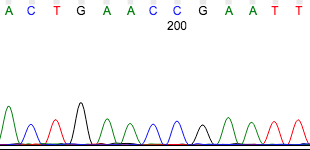


M30-MT1_FOR3 (Allele detected: A)


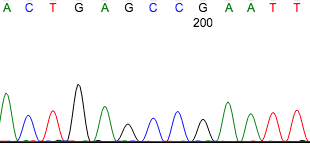


M31-MT1_FOR3 (Allele detected: G)


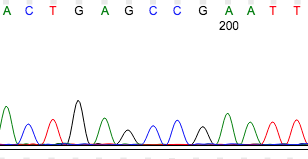


M32-MT1_FOR3 (Allele detected: G)


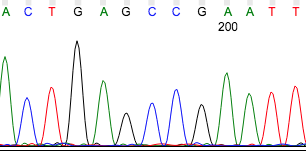


M33-MT1_FOR3 (Allele detected: G)


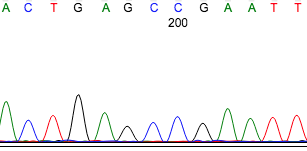


M34-MT1_FOR3 (Allele detected: G)


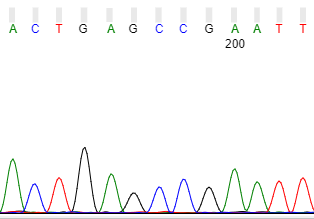


M35-MT1_FOR3 (Allele detected: G)


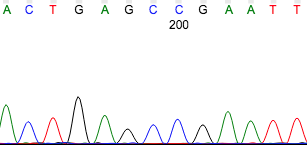


M36-MT1_FOR3 (Allele detected: G)


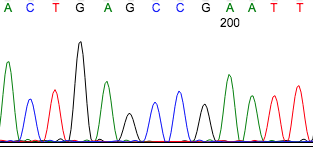


M37-MT1_FOR3 (Allele detected: G)


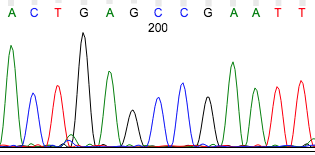


M38-MT1_FOR3 (Allele detected: G)


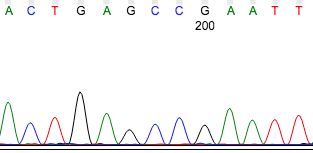


M39-MT1_FOR3 (Allele detected: G)


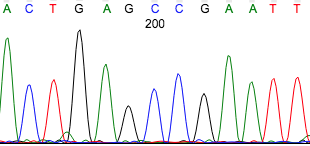


M40-MT1_FOR3 (Allele detected: G)


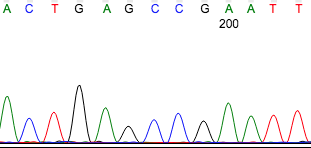


M41-MT1_FOR3 (Allele detected: G)


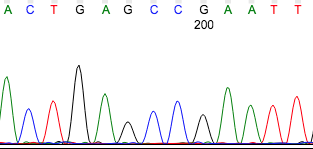


M42-MT1_FOR3 (Allele detected: G)


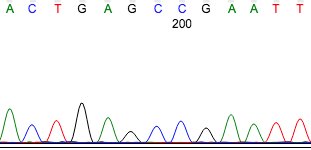


M43-MT1_FOR3 (Allele detected: G)


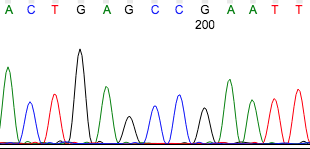


M44-MT1_FOR3 (Allele detected: G)


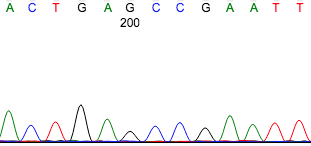


M45-MT1_FOR3 (Allele detected: G)


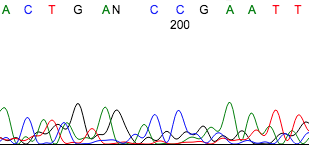


M46-MT1_FOR3 (Allele detected: G, Poor quality)


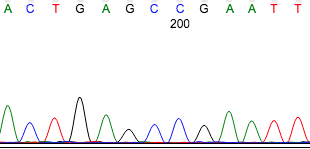


M47-MT1_FOR3 (Allele detected: G)


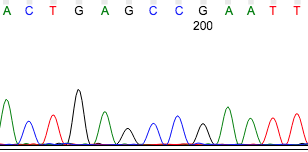


M48-MT1_FOR3 (Allele detected: G)


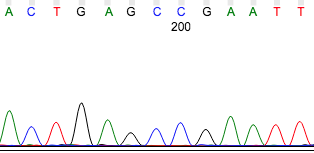


M49-MT1_FOR3 (Allele detected: G)


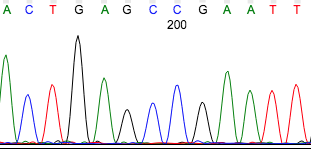


M50-MT1_FOR3 (Allele detected: G)


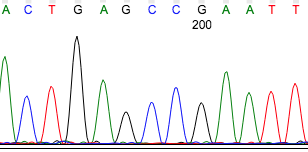


M51-MT1_FOR3 (Allele detected: G)


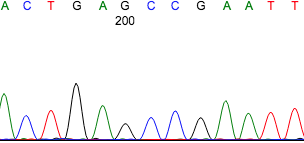


M52-MT1_FOR3 (Allele detected: G)


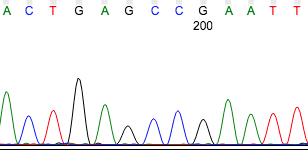


M53-MT1_FOR3 (Allele detected: G)


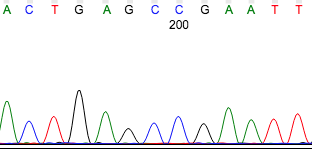


M54-MT1_FOR3 (Allele detected: G)


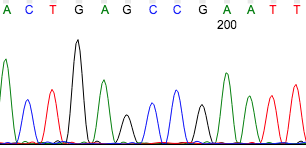


M55-MT1_FOR3 (Allele detected: G)


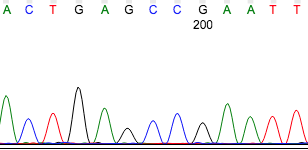


M56-MT1_FOR3 (Allele detected: G)


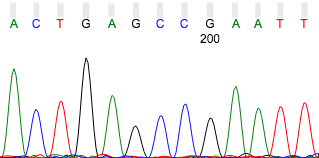


M57-MT1_FOR3 (Allele detected: G)


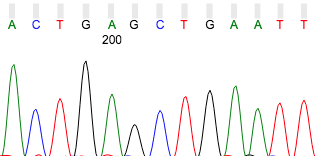


M58-MT1_FOR3 (Allele detected: GCT)


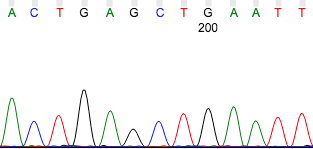


M59-MT1_FOR3 (Allele detected: GCT)


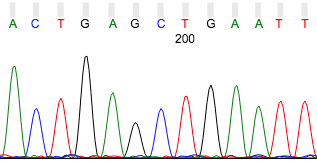


M60-MT1_FOR3 (Allele detected: GCT)

## Figure S3


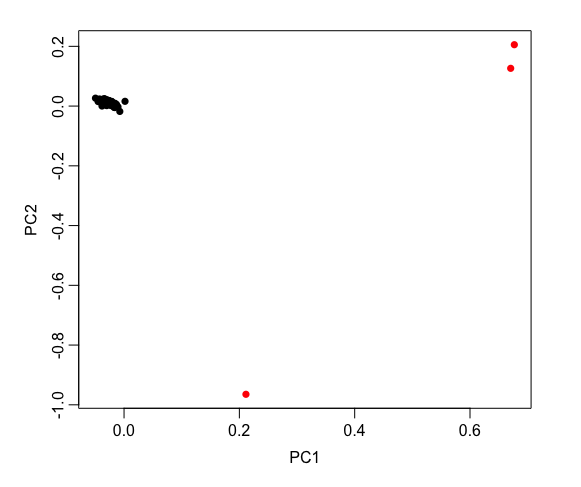


Principal component analysis using the genomic SNPs extracted for each sample. The first 2 principal components (PC1 and PC2) are depicted. The outlier samples are indicated in red.
